# Hospital anxiety and depression after ICU survival: results of a post-ICU aftercare program

**DOI:** 10.1186/cc14632

**Published:** 2015-03-16

**Authors:** D Ramnarain, C Slobbe, W Schapendonk, J Van Gorp, I Gnirrip, S Voermans, A Rutten, G Van der Nat, N Van der Lely

**Affiliations:** 1 St.Elisabeth Hospital Tilburg, the Netherlands

## Introduction

Although the ICU survival rate has increased in the last decade, the negative effects on mental health and related quality of life become more clear. In the literature the prevalence of anxiety and depressive symptoms post ICU ranges from 10 to 43% [1]. Early recognition and treatment of anxiety and depressive symptoms is important because depression caries a risk for suicide, limited quality of life, and delayed return to work. We studied hospital anxiety and depression (HAD) symptoms after ICU discharge.

## Methods

Patients who were treated in our ICU from 1 January 2013 until 31 December 2013 for more than 5 days were invited to visit our post-ICU aftercare clinic. Six weeks after discharge they received a letter of invitation together with a health-related questionnaire, the Hospital Anxiety and Depression Scale (HADS) questionnaire [2]. Patients were asked to return the questionnaire prior to their visit. All data were analyzed and if the HADS score indicated a clinically significant anxiety or depression, patients were referred to a psychologist for further analyses and treatment. All patient data were analyzed retrospectively.

## Results

Seventy-nine patients, 54 men and 43 women, mean age 57 years. Median APACHE II and IV was 18 and 60 respectively. Median ICU and hospitals days were 9 and 20 respectively. Seventy-six percent were mechanically ventilated with a median of 5 days. Median time after ICU discharge to aftercare visit was 165 days. Patients were divided into three categories: 1, no HAD (45.4%); 2, possible HAD (9.3%); and 3, clinically significant HAD (45.4%). Women compared with men showed significantly more HAD symptoms (26.8% vs. 18.6%, *P *< 0.05). Patients with subarachnoid hemorrhage, neurotrauma and multitrauma patients showed more HAD symptoms. Pain, fatigue, muscle weakness, impairment of daily activity dyspnea, and hoarseness were significantly associated with clinically significant HAD. No association between age and HAD was found. Diagnosis at ICU admission, length of stay, severity of illness, delirium and use of sedatives were not associated with HAD.

## Conclusion

Prevalence of clinically significant post-ICU HAD was 45.4%. Female sex and post-ICU physical complaints - pain, fatigue, muscle weakness, impairment in daily activities, hoarseness and dyspnea - were significantly associated with HAD.
